# Alcohol and tobacco consumption concordance and its correlates in older couples in Latin America

**DOI:** 10.1111/ggi.12974

**Published:** 2017-01-06

**Authors:** Mayra Pires Alves Machado, Davi Camara Opaleye, Tiago Veiga Pereira, Ivan Padilla, Ana Regina Noto, Martin Prince, Cleusa Pinheiro Ferri

**Affiliations:** ^1^ Universidade Federal de Sao Paulo Department of Psychobiology Sao Paulo Brazil; ^2^ Hospital Alemao Oswaldo Cruz Institute of Education and Health Sciences Sao Paulo Brazil; ^3^ Centre for Global Mental Health, Health Service and Population Research Department King's College London, Institute of Psychiatry, Psychology and Neuroscience London UK

**Keywords:** aging, concordance, elderly, health behavior, spouses

## Abstract

**Aim:**

As little is known about alcohol and tobacco consumption concordance between older spouses in low‐ and middle‐income countries, the present study aimed to estimate this in older couples from five Latin American countries.

**Methods:**

This study is a secondary analysis of data collected between 2003 and 2007 by the 10/66 Dementia Research Group, from 1451 couples aged over 65 years from Cuba, the Dominican Republic, Peru, Mexico and Puerto Rico. Kappa statistic was used to assess the agreement of the behavior beyond chance, and logistic regression models with meta‐analyses were used to estimate the factors associated with concordance.

**Results:**

The mean age of the total sample was 74.8 years (SD 6.6). The results showed high levels of agreement rates in relation to drinking and smoking (75.9% and 85% of couples, respectively, did not drink or smoke), which were beyond the agreement expected by chance. Increased age was associated with concordance on both being non‐drinkers (OR 1.03, 95% CI 1.01–1.05) and non‐smokers (OR 1.05, 95% CI 1.02–1.07); and having a larger social network was associated with less likelihood of the couple being non‐drinkers (OR 0.93, 95% CI 0.88–0.98). Attending religious meetings was associated with increased likelihood of the couple being non‐smokers (OR 1.19, 95% CI 1.01–1.41). Socioeconomic circumstances were not associated with couples’ concordance.

**Conclusions:**

Older Latin American couples have high levels of concordance in drinking and smoking habits, which increases with age, and were not associated with socioeconomic circumstances, but were with social network. This knowledge can assist the development of policies and interventions to promote health among this growing population. **Geriatr Gerontol Int 2017; 17: 1849–1857**.

## Introduction

Globally, the proportion of older people is sharply increasing, particularly in low‐ and middle‐income countries, placing pressure on health service provision.^1^ This process is accompanied by an increase in the burden of chronic conditions, such as cardiovascular diseases, cancer, diabetes and chronic respiratory illness, which are already responsible for 70% of deaths among people aged 70 years or older, in addition to causing disabilities and suffering for many more.^2^ Treating chronic diseases is expensive, but they can be prevented, or at least the risk lessened by lifestyle changes.^2^

In general, being married seems to bring health benefits, and married people might live longer and with fewer physical limitations.^3^, ^4^ Although the mechanisms for these benefits are not fully understood, one important element seems to be the positive influence spouses have on each other's health behaviors.^5^, ^6^ Most studies on this have concentrated on the earlier phases of marriage.^7^ However, two studies with older couples, one carried out in the USA^8^ using data from the Health and Retirement Study and the other in the UK,^9^ have examined how healthy behavior in one spouse tends to have a positive influence on the other. The influence one spouse has on the other has been examined in relation to other attitudes and behaviors using data from the Health and Retirement Study in the USA. Drewelies *et al*. found that having a partner with higher levels of self‐efficacy was associated with fewer functional limitations, better self‐rated health and more physical activity.^10^ One spouse's level of optimism in older couples was also found to have a positive influence on their partner's health, regardless of their own level of optimism.^11^ Regarding drinking behavior specifically, another study also using data from the Health and Retirement Study in the USA showed that couples in which both spouses drank reported decreased negative marital quality, and that this effect was greater for wives than husbands.^12^ These studies focused on older couples, but only in high‐income countries, and very little is currently known about older couples’ health behavior concordance in low‐ and middle‐income countries, where aging is occurring at a rapid rate. To address this knowledge gap, we used population‐based studies on older people from Cuba, the Dominican Republic, Mexico, Puerto Rico and Peru to estimate the concordance in couples for two important health behaviors (drinking and smoking), tested if these rates were beyond chance and examined potential factors associated with these concordances.

## Methods

### Participants, settings and procedures

This was a secondary analysis of the cross‐sectional phase of the 10/66 population‐based study that was carried out from 2003 to 2007. It comprised all people aged 65 years and older living in geographically‐defined catchment areas in China, Cuba, the Dominican Republic, India, Mexico, Puerto Rico, Peru and Venezuela. The response rates were above 80% in all countries. More details of the study can be found in a previous publication.^13^

For the present analysis, we selected data from the Latin American countries (Cuba, the Dominican Republic, Peru, Mexico and Puerto Rico, *n* = 10 900) except Venezuela, which was excluded because of missing data regarding alcohol consumption (36%). We then identified all married participants (*n* = 4924). From the total of married participants, we were able to identify with certainty 2902 participants (1451 couples) who lived in the same household and were married to each other.

### Measures

We obtained information on age, educational level, number of household assets and whether the participants were in receipt of any income, benefits, pensions or allowances. When they did receive any income, they were asked to specify the type, including: government pension, occupational pension, disability pension or benefit, money from family, income from rented land or property, income from paid work, or other. We also estimated the extent of each individual's social network. The total score for this item was composed by scores given to the participants’ self‐reported frequency (never, occasionally or regularly) of each one of the following social activities: attending religious meetings, attending any community or social groups, seeing children and relatives, and having a chat or any sort of social activities with friends or neighbors. The total score varied from 0 to 10, and was categorized as follows: 0–3 “small social network”; 4–6 “moderate social network”; and 7–10 “large social network”.

The number of impairments was determined by a sum of self‐reported physical impairments that interfered with the participants’ daily activities.^14^

Physical activity: Participants were asked about their self‐perception of being physically active, taking into account both work and leisure. Those answering “very” or “fairly” were categorized as physically active, and those who answered “not very” or “not at al” as physically inactive.

Alcohol consumption: Data on the amount and frequency of maximum regular consumption of standard alcohol units in an average week were gathered by self‐report and categorized according to guidelines for safe drinking (no more than 7 units per week).^15^ We first identified two patterns of alcohol consumption: (i) no drinking; and (ii) any drinking (one or more units per week). Then we classified the any drinkers group into two other patterns of alcohol consumption, also according to the same guidelines: (i) moderate drinkers (between 1 and 7 units per week); and (ii) at risk drinkers (8 or more units per week).^15^

Smoking: Data were collected by the participants’ self‐report on regular use of tobacco.

### Statistical analysis

Agreement within couples: For each health behavior, the kappa statistic was used to assess the agreement of wife and husband beyond chance. We estimated 95% confidence intervals using 100 000 bootstrap replications.^16^ However, as the prevalence of the health behavior influences the kappa statistic, we calculated the prevalence‐adjusted bias‐adjusted kappa (PABAK).^17^ We followed the interpretation of PABAK proposed by Landis and Koch^18^: <0 = poor; 0–0.20 = slight; 0.21–0.40 = fair; 0.41–0.60 = moderate; 0.61–0.80 = substantial; and 0.81–1.00 = almost perfect agreement. We also applied McNemar's test to verify the symmetry of the discordant couples, making explicit allowance for the dependency in the data generated by the marriage. This test assumes that the proportion of discordant pairs *b* (wife = Yes, husband = No) and *c* (wife = No, husband = Yes) should be equal under the null hypothesis.

Factors associated with health behavior concordance: We used logistic regression to investigate how women and men's age, schooling, household assets, receipt of any income, width of social network, number of physical impairments and physical activity were associated with health behavior concordances (full model adjusted for all variables assessed and also for couple identification clustering). In order to account for the clustering effect, robust standard errors were used instead. This “sandwich estimator of variance” relaxes the premise that observations are independent. Hence, logistic regression‐based concordance analyses employed a total of 2902 participants. However, standard errors for these analyses were adjusted for 1451 clusters.

We carried out a meta‐analysis using a two‐stage process. First, we analyzed individual participant data separately in each country to produce country‐specific estimates. These analyses were based on the “full model,” and incorporated the aforementioned sandwich estimator for the variance. Then, a summary, overall estimate was calculated as a weighted average of the country‐specific estimates using both fixed‐ and random‐effects models (general inverse variance and DerSimonian–Laird methods,^19^ respectively). Fixed‐effects models assume that there is a common effect size across countries (e.g. the strength of the association is identical across studies), and that any observed variability in the estimates is as a result of sampling error only. In contrast, random‐effects models consider that there might be different strengths of association across countries, and incorporate the between‐country variability in the calculations – usually providing wider confidence intervals. Statistical heterogeneity was tested using the Cochran's *Q*‐test and quantified with the index *I* ^*2*^.^20^ Throughout our analysis, results with a *P* < 0.05 were considered statistically significant, except for the *Q*‐test, which was considered statistically significant when *P* < 0.10.^21^

We also carried out a sensitivity analysis in which we used the full‐adjusted model with all the components of the social network separately, in order to estimate if specific social activities were associated with health behavior concordance. We carried out the analysis separately for each country, and then summarized each into a single estimate using a meta‐analysis.

## Results

### General characteristics of the participants

A general description of the individuals by each country and sex is given in Table 1. There was a higher proportion of women in the younger age groups (33.9% of the total sample were aged between 65 and 69 years) compared with men (14.6%), and a higher proportion of men in the older age group (31.9% were aged 80 and older) compared with women (15.2%), which was similar in all countries. The mean age for women and men was also similar in all countries, and not much different from the overall mean age for each sex, being 73.0 years (SD 6.0) for women and 76.5 years (SD 6.7) for men.

**Table 1:** Characteristics of the sample by sex and country

	Mexico		Cuba		Dominican Republic		Puerto Rico		Peru		Total	
	*n* = 598		*n* = 770		*n* = 338		*n* = 704		*n* = 492		*n* = 2902	
	*n* (%)		*n* (%)		*n* (%)		*n* (%)		*n* (%)		*n* (%)	
	Female	Male	Female	Male	Female	Male	Female	Male	Female	Male	Female	Male
Age (years)												
Mean age (SD)	71.9 (5.4)	76.0 (5.6)	72.9 (5.6)	75.7 (6.3)	71.8 (4.9)	76.8 (7.1)	74.7 (6.5)	77.8 (7.1)	73.0 (6.6)	76.6 (7.3)	73.0 (6.0)	76.5 (6.7)
Min–max	65–88	65–96	65–89	65–94	65–87	65–99	65–99	65–99	65–97	65–101	65–99	65–101
Age group (years)												
65–69	122 (40.8)	36 (12.1)	130 (33.8)	66 (17.1)	59 (34.9)	28 (16.6)	88 (25.0)	40 (11.4)	93 (37.8)	42 (17.1)	492 (33.9)	212 (14.6)
70–74	90 (30.1)	94 (31.4)	114 (29.6)	115 (29.9)	64 (37.9)	46 (27.2)	94 (26.7)	83 (23.6)	59 (24.0)	67 (27.2)	421 (29.0)	405 (27.9)
75–79	49 (16.4)	90 (30.1)	87 (22.6)	97 (25.2)	37 (21.9)	36 (21.3)	92 (26.1)	92 (26.1)	50 (20.3)	54 (21.9)	315 (21.7)	369 (25.4)
80+	38 (12.7)	79 (26.4)	52 (13.5)	105 (27.3)	9 (5.3)	59 (34.9)	78 (22.2)	137 (38.9)	44 (17.9)	83 (33.8)	221 (15.2)	463 (31.9)
Missing	0 (0.0)	0 (0.0)	2 (0.5)	2 (0.5)	0 (0.0)	0 (0.0)	0 (0.0)	0 (0.0)	0 (0.0)	0 (0.0)	2 (0.2)	2 (0.2)
Education												
None or minimal	217 (72.6)	216 (72.2)	80 (20.8)	55 (14.3)	119 (70.4)	113 (66.8)	69 (19.6)	56 (15.9)	43 (17.5)	23 (9.4)	528 (36.4)	463 (31.9)
Primary completed	48 (16.0)	49 (16.4)	132 (34.3)	127 (33.0)	30 (17.8)	37 (21.9)	84 (23.8)	68 (19.3)	85 (34.5)	96 (39.0)	379 (26.1)	377 (26.0)
Secondary completed	23 (7.7)	13 (4.4)	109 (28.3)	118 (30.6)	10 (5.9)	15 (8.9)	121 (34.4)	150 (42.6)	80 (32.5)	76 (30.9)	343 (23.6)	372 (25.6)
Tertiary	11 (3.7)	21 (7.0)	64 (16.6)	85 (22.1)	8 (4.7)	3 (1.8)	77 (21.9)	77 (21.9)	38 (15.5)	51 (20.7)	198 (13.7)	237 (16.3)
Missing	0 (0.0)	0 (0.0)	0 (0.0)	0 (0.0)	2 (1.2)	1 (0.6)	1 (0.3)	1 (0.3)	0 (0.0)	0 (0.0)	3 (0.2)	2 (0.2)
Household assets (couple)												
0–2	50 (8.4)		4 (0.5)		12 (3.5)		2 (0.3)		9 (1.8)		77 (2.6)	
3–5	197 (32.9)		199 (25.9)		149 (44.1)		6 (0.8)		92 (18.7)		643 (22.2)	
6–7	351 (58.7)		563 (73.1)		176 (52.1)		696 (98.9)		391 (79.5)		2177 (75.0)	
Missing	0 (0.0)		4 (0.5)		1 (0.3)		0 (0.0)		0 (0.0)		5 (0.2)	
Any income												
Yes	202 (67.6)	253 (84.6)	242 (62.9)	376 (97.6)	111 (65.7)	132 (78.1)	340 (96.6)	342 (97.2)	133 (54.1)	210 (85.4)	1028 (70.9)	1313 (90.5)
Missing	0 (0.0)	0 (0.0)	2 (0.5)	1 (0.3)	4 (2.4)	0 (0.0)	4 (1.1)	5 (1.4)	5 (2.0)	3 (1.2)	15 (1.0)	9 (0.6)
Network score												
Low (0–3)	33 (11.1)	30 (10.0)	21 (5.4)	19 (4.9)	1 (0.6)	6 (3.5)	30 (8.5)	30 (8.5)	10 (4.1)	12 (4.9)	95 (6.6)	97 (6.7)
Moderate (4–6)	155 (51.8)	144 (48.2)	202 (52.5)	197 (51.1)	33 (19.5)	46 (27.2)	146 (41.5)	165 (46.9)	45 (18.3)	56 (22.8)	581 (40.0)	608 (41.9)
Wide (7–10)	110 (36.8)	123 (41.1)	162 (42.1)	169 (43.9)	131 (77.5)	113 (66.9)	176 (50.0)	157 (44.6)	183 (74.4)	173 (70.3)	762 (52.5)	735 (50.6)
Missing	1 (0.3)	2 (0.7)	0 (0.0)	0 (0.0)	4 (2.4)	4 (2.4)	0 (0.0)	0 (0.0)	8 (3.2)	5 (2.0)	13 (0.9)	11 (0.8)
No. impairments												
0	91 (30.4)	103 (34.4)	123 (32.0)	153 (39.7)	42 (24.8)	43 (25.4)	81 (23.0)	93 (26.4)	92 (37.4)	99 (40.3)	429 (29.5)	491 (33.8)
1–2	139 (46.5)	138 (46.2)	198 (51.4)	176 (45.7)	76 (45.0)	81 (47.9)	169 (48.0)	155 (44.0)	110 (44.7)	111 (45.1)	692 (47.7)	661 (45.6)
3‐more	69 (23.1)	57 (19.1)	64 (16.6)	50 (13.0)	49 (29.0)	42 (24.9)	101 (28.7)	103 (29.3)	43 (17.5)	35 (14.2)	326 (22.5)	287 (19.8)
Missing	0 (0.0)	1 (0.3)	0 (0.0)	6 (1.6)	2 (1.2)	3 (1.8)	1 (0.3)	1 (0.3)	1 (0.4)	1 (0.4)	4 (0.3)	12 (0.8)
Health behavior												
Moderate drinking	31 (10.4)	69 (23.1)	11 (2.9)	44 (11.4)	6 (3.5)	15 (8.9)	5 (1.4)	58 (16.5)	2 (0.8)	17 (6.9)	55 (3.8)	203 (14.0)
At‐risk drinking	1 (0.3)	27 (9.0)	9 (2.3)	40 (10.4)	9 (5.3)	42 (24.8)	3 (0.8)	29 (8.2)	0 (0.0)	3 (1.2)	22 (1.5)	141 (9.7)
Smoking	6 (2.0)	45 (15.0)	37 (9.6)	93 (24.1)	16 (9.5)	20 (11.8)	4 (1.1)	26 (7.4)	5 (2.0)	18 (7.3)	68 (4.7)	202 (13.9)
Physical activity												
Yes	214 (71.6)	190 (63.5)	261 (67.8)	286 (74.3)	116 (68.6)	108 (63.9)	232 (65.9)	223 (63.3)	183 (74.4)	161 (65.4)	1006 (69.3)	968 (66.7)
No	82 (27.4)	108 (36.1)	124 (32.2)	99 (25.7)	53 (31.3)	59 (34.9)	119 (33.8)	129 (36.6)	63 (25.6)	84 (34.1)	441 (30.4)	479 (33.0)

There was a high proportion of participants with no or minimal education in Mexico and the Dominican Republic (approximately 70% for both sexes), and only a small proportion of participants with tertiary education in all countries. A higher proportion of men were receiving some kind of income (90.5% of all male participants) compared with women (70.9% of all female participants) in all countries. A total of 75% of the sample had more than five household assets. Regarding the participant's social network, nearly 50% of the total sample had a low or moderate social network. There was a slightly higher proportion of men without any physical impairment (33.8% *vs* 29.5% of the total sample), and a slightly higher proportion of women with a higher number of physical impairments.

The overall prevalence of moderate drinking among women was 3.8% (ranging from 0.8% in Peru to 10.4% in Mexico), and among men was 14% (ranging from 6.9% in Peru to 23.1% in Mexico). There was a higher proportion of at‐risk drinking in the Dominican Republic (5.3% of women and 24.8% of men) and, in general, at‐risk drinking was higher among men (9.7% of the total sample) than women (1.5% of the total sample). Overall, smoking was reported by 4.7% of the women (ranging from 1.1% in Puerto Rico to 9.6% in Cuba), and 13.9% of the men (ranging from 7.3% in Peru to 24.1% in Cuba). There was also a higher proportion of male former smokers (44.2%) than female former smokers (12.6%), ranging from 7.4% in Mexico to 22.5% in the Dominican Republic for women, and from 19.9% in Peru to 58.6% in the Dominican Republic for men. Regarding physical activity, there was a higher prevalence of physically active participants (66.7% for men and 69.3% for women) compared with physically inactive participants (33% for men and 30.4% for women). Overall, men were more inactive than women, with the exception of Cuba (32.2% of women compared with 25.7% of men).

### Concordance of wife and husband for alcohol consumption

Overall, non‐drinking behavior showed a high level of agreement (75.9%) between wife and husband. This agreement was 3% higher than that which would be expected by chance (*P* < 0.001; Table 2). PABAK estimates ranged from 0.24 (Dominican Republic) to 0.83 (Peru), suggesting fair to substantial agreement for alcohol consumption, respectively. Similar results were observed for moderate drinking, as well as for the “at‐risk drinking.” Among discordant couples, non‐agreement was asymmetrical, indicating that couples in which the husband drinks, but the wife does not, are sevenfold more common (OR 7.3, 95CI 5.16–10.21) than couples in an inverse situation (wife drinks and husband does not).

**Table 2:** Prevalence of health behavior concordance among spouses

Country	*n*	Both spouses *n* (%)	Men only *n* (%)	Women only *n* (%)	Neither spouse *n* (%)	Obs. Agreem.**†** (%)	Exp. Agreem.**‡** (%)	Kappa		PABAK		*P*
								Coef.**§**	(95%CI)	Coef.**§**	(95%CI)	
**Alcohol drinking concordance: any drinking**												
Mexico	295	17 (5.8)	79 (26.8)	15 (5.1)	184 (62.3)	68.1	63.6	0.12	(0.02–0.23)	0.36	(0.25–0,46)	<0.001
Cuba	371	13 (3.5)	70 (18.9)	7 (1.9)	281 (75.7)	79.2	74.6	0.18	(0.08–0.29)	0.58	(0.50–0.66)	<0.001
Dominican Rep.¶	165	5 (3.0)	52 (31.5)	10 (6.1)	98 (59.4)	62.4	62.6	‐0.01	(−0.11–0.11)	0.24	(0.10–0.39)	0.541
Puerto Rico	350	3 (0.9)	84 (24.0)	5 (1.4)	258 (73.7)	74.5	73.9	0.02	(−0.03–0.09)	0.49	(0.39–0.58)	0.2013
Peru	235	1 (0.4)	18 (7.7)	1 (0.4)	215 (91.5)	91.9	91.2	0.08	(−0.01–0.30)	0.83	(0.76–0.90)	0.015
Overall	1416	39 (2.7)	303 (21.4)	38 (2.6)	1036 (73.1)	75.9	73.0	0.10	(0.06–0.15)	0.51	(0.47–0.56)	<0.001
**Alcohol drinking concordance: moderate drinking**												
Mexico	295	14 (4.8)	55 (18.6)	17 (5.8)	209 (70.8)	75.5	71.0	0.16	(0.04–0.28)	0.51	(0.41–0.60)	0.001
Cuba	371	7 (1.9)	36 (9.7)	4 (1.1)	324 (87.3)	89.2	86.1	0.22	(0.07–0.38)	0.78	(0.72–0.84)	<0.001
Dominican Rep.¶	165	3 (1.8)	12 (7.3)	3 (1.8)	147 (89.1)	90.9	87.9	0.24	(−0.02–0.52)	0.81	(0.73–0.90)	<0.001
Puerto Rico	350	2 (0.6)	56 (16.0)	3 (0.8)	289 (82.6)	83.1	82.5	0.04	(−0.02–0.13)	0.66	(0.58–0.74)	0.078
Peru	235	1 (0.4)	16 (6.8)	1 (0.4)	217 (92.4)	92.7	92.0	0.09	(−0.01–0.32)	0.85	(0.78–0.92)	0.009
Overall	1416	27 (1.9)	175 (12.4)	28 (2.0)	1186 (83.7)	85.6	82.9	0.16	(0.09–0.22)	0.71	(0.67–0.74)	<0.001
**Alcohol drinking concordance: at risk drinking**												
Mexico	295	0 (0.0)	27 (9.1)	1 (0.3)	267 (90.6)	99.5	99.5	‐0.01	(−0.02–0.00)	0.81	(0.79–0.82)	0.624
Cuba	371	(4 (1.1)	36 (9.7)	5 (1.3)	326 (87.9)	88.9	87.3	0.13	(0.01–0.28)	0.77	(0.71–0.84)	<0.001
Dominican Rep.¶	165	2 (1.2)	40 (24.3)	7 (4.2)	116 (70.3)	71.5	71.8	‐0.01	(−0.01–0.11)	0.43	(0.29–0.56)	0.590
Puerto Rico	350	0 (0.0)	29 (8.3)	3 (0.8)	318 (90.9)	90.8	91.0	‐0.01	(−0.03–0.00)	0.81	(0.75–0.87)	0.699
Peru	235	0 (0.0)	2 (0.8)	0 (0.0)	233 (99.2)	99.1	99.1	0.000	.	0.98	(0.95–1.00)	.
Overall	1416	6 (0.4)	134 (9.5)	16 (1.1)	1260 (89.0)	89.4	88.8	0.05	(0.00–0.11)	0.78	(0.75–0.82)	0.003
**Smoking concordance**												
Mexico	299	3 (1.0)	42 (14.0)	3 (1.1)	251 (83.9)	84.9	83.5	0.08	(−0.01–0.22)	0.69	(0.61–0.77)	0.008
Cuba	385	19 (4.9)	74 (19.2)	18 (4.7)	274 (71.2)	76.1	70.8	0.18	(0.07–0.29)	0.52	(0.43–0.60)	<0.001
Dominican Rep.¶	169	4 (2.4)	16 (9.5)	12 (7.1)	137 (81.0)	83.4	80.9	0.13	(−0.04–0.34)	0.66	(0.55–0.78)	0.043
Puerto Rico	352	0 (0.0)	26 (7.4)	4 (1.1)	322 (91.5)	91.4	91.6	‐0.02	(−0.04–0.00)	0.82	(0.77–0.88)	0.715
Peru	246	0 (0.0)	18 (7.3)	5 (2.1)	223 (90.6)	90.6	90.9	‐0.03	(−0.05–0.01)	0.81	(0.74–0.88)	0.737
Overall	1451	26 (1.8)	176 (12.1)	42 (2.9)	1207 (83.2)	85.0	82.7	0.13	(0.07–0.19)	0.70	(0.66–0,73)	<0.001

† Observed agreement.

‡ Expected agreement.

§ Coefficient.

¶ Dominican Republic.

### Concordance of wife and husband for smoking

Considering all the countries together, agreement for non‐smoking status was also high (85%), with an overall PABAK of 0.70, indicating substantial agreement between wife and husband (Table 2). The overall observed agreement was 2% higher than the agreement expected by chance alone (*P* < 0.001). For the discordant couples, non‐agreement for smoking was also asymmetrical, showing that discordant couples were fourfold more likely to comprise a smoker husband and a non‐smoker wife (OR 4.13, 95% CI 2.06–82.7) than a non‐smoker husband and a smoker wife.

### Factors associated to concordance between wife and husband

We also identified the characteristics of spouses potentially associated with healthy behavior concordance, which were: both being non‐drinkers and both being non‐smokers (see Table 3). Increased age was associated with a higher likelihood of spousal concordance on both being non‐drinkers (OR 1.03, 95% CI 1.01–1.05 – heterogeneity test 4.99 [4], *P* = 0.288; *I* ^2^ = 20 [0–83]) and non‐smokers (OR 1.05, 95% CI 1.02–1.07 – heterogeneity test 4.44 [4], *P* = 0.349; *I* ^2^ = 10 [0–81]). Furthermore, the fixed‐effects model showed that the wider the social network, the smaller the chance of both spouses being non‐drinkers (OR 0.93, 95% CI 0.88–0.98 – heterogeneity test 7.08 [4], *P* = 0.132; *I* ^2^ = 44 [0–79]), which was not seen in the random‐effect model (OR 0.93, 95% CI 0.87–1.01).

**Table 3:** Correlates of couple concordance on alcohol and tobacco consumption (pooled odds ratios from meta‐analysis of country estimates)

	Both non‐drinkers		Both non‐smokers	
	OR**†**	(95% CI)	OR**†**	(95% CI)
**Age**				
	1.03	(1.01–1.05)	1.05	(1.02–1.07)
**Educational level**				
	1.04	(0.93–1.15)	1.11	(0.98–1.26)
**In receipt of any income**				
	0.96	(0.73–1.26)	1.05	(0.79–1.40)
**Household assets**				
	1.05	(0.94–1.17)	1.00	(0.88–1.14)
**Social network**				
Total	0.93	(0.87–0.98)	1.04	(0.97–1.10)
Attend religious meetings	0.99	(0.83–1.19)	1.19	(1.01–1.41)
Attend social meetings	0.86	(0.77–0.97)	1.06	(0.92–1.23)
See children or relatives	1.20	(0.98–1.47)	1.05	(0.83–1.32)
Chat with friends	0.84	(0.74–0.94)	1.00	(0.86–1.16)
See neighbors	0.79	(0.66–0.94)	1.04	(0.85–1.28)
**No. impairments**				
	1.03	(0.90–1.18)	1.00	(0.88–1.13)
**Physical activity**	0.97	(0.87–1.08)	0.97	(0.85–1.11)

† Pooled odds ratio adjusted by sex, age, education, in receipt of any income, assets, social network, number of impairments, physical activity, and alcohol and tobacco consumption accordingly, plus couple identification clustering.

In order to better understand the association between social network and health behavior concordance, we analyzed each of the five social activities separately. Non‐drinking concordance was inversely associated with attending social meetings (OR 0.86, 95% CI 0.77–0.97 – heterogeneity test 4.01 [4], *P* = 0.405; *I* ^2^ = 0 [0–79]), chatting with friends (OR 0.84, 95% CI 0.74–0.94 – heterogeneity test 1.27 [4], *P* = 0.867; *I* ^2^ = 0 [0–79]) and seeing neighbors (OR 0.79, 95% CI 0.66–0.94 – heterogeneity test 2.50 [3] *P* = 0.476; *I* ^2^ = 0 [0–85]). The more the individual took part in these activities, the smaller was the chance of both spouses being non‐drinkers. In contrast, attending religious meetings was positively associated with non‐smoking concordance (OR 1.19, 95% CI 1.01–1.41 – heterogeneity test 3.36 [4], *P* = 0.500; *I* ^2^ = 0 [0–79]).

## Discussion

It seems that a significant proportion of older couples in Latin America are concordant in their non‐drinking and non‐smoking habits, and when discordant the most common status is that the husband smokes or drinks and the wife does not. The present findings regarding rates of alcohol non‐drinking concordance are not consistent with some previous studies carried out in developed countries. Graham *et al*., for example, found higher rates of drinking concordance (47.4% compared with 2.7% in the present study) and lower rates of non‐drinking concordance (26.8% compared with 73%) among a community sample of older adults in Canada.^22^ A more recent study, using a sample of older couples in the USA^12^ found similar rates as Graham *et al*.,^22^ in which 45% were drinking concordant and 29% were non‐drinking concordant. Nevertheless, the Canadian study's rates of concordance on smoking and non‐smoking were similar to those found in the present study. These differences regarding alcohol consumption among couples between the present study and other studies, as well as the similarities between studies regarding the concordance on tobacco consumption, might just be a reflection of the situation in terms of tobacco and alcohol consumption in high‐income countries, and low‐ and middle‐income countries. It seems that alcohol consumption by older individuals in Western high‐income countries is more common than in low‐ and middle‐income countries. Hajat *et al*. found that alcohol consumption was much higher in the UK, with 73% of people aged 75 years and older being moderate drinkers, compared with the 8.9% in the present study.^23^ However, they found that 9.8% of people aged over 75 years in the UK were smokers, a similar proportion to the 9.3% of smokers aged over 65 years in the present study. These differences might be related to the economic and developmental states of the countries, as well as to the price and availability of alcohol and tobacco and country policies. Local cultural issues related to drinking and smoking behavior might also play a role, and are likely to be important in explaining the differences found in the prevalence of these behaviors among older people between the countries in our study.

Another factor that could play an important role in the differences between the present study and other studies is the prevalence of chronic health conditions. Previous studies were carried out in high‐income countries, where the prevalence of chronic diseases and disability are lower than those found in developing countries. Chronic conditions might affect behavior change, in the sense that one might stop smoking or drinking as a result of chronic conditions, such as hypertension or diabetes, which could partially explain the lower prevalence of alcohol drinking among older adults in Latin America, which is in turn reflected in the low proportion of concordance on drinking behavior among older spouses in the present study compared with older spouses from high‐income countries.^24^, ^25^ However, when we consider tobacco consumption prevalence, older adults’ tobacco consumption and smoking habits and the concordance found in older couples in the present study are similar to those in studies carried out in high‐income countries. Unlike in the case of alcohol, tobacco control polices have being carried out with relative success in many countries, including those in Latin America. According to the World Health Organization report on the global tobacco epidemic, these policies have been implemented in both high‐ and low‐ and middle‐income countries, and have decreased the global prevalence of smoking from 23% in 2007 to 21% in 2013.^26^ The lower rates of tobacco consumption in general might be reflected in the high levels of concordance found in the present study and in studies carried out in richer countries.

The decline in health with age could also explain the association found between age and concordance between spouses in terms of being non‐drinkers and non‐smokers, whereby the older the spouses are, the more likely they are to be non‐drinkers and non‐smokers.^23^, ^27^ However, regardless of their age or number of impairments, we found that the wider the social network, the lower was the probability that both spouses were non‐drinkers. Close social networks, such as family (seeing children or close relatives) and religious activities, were not associated with spouse concordance on alcohol consumption, whereas social networks made up of those with whom the spouses had less close relationships (attending social meetings, having a chat with friends and seeing neighbors) were associated with concordance. Having a wider social network is known to be a factor associated with alcohol consumption, and according to Rosenquist *et al*., the closer the social contact was, such as a spouse or close friend or relative, the more similar the patterns of alcohol consumption were.^28^ However, this influence was moderated by geographic distance. Although a previous study showed that closer social contacts also influenced smoking abstinence in former smokers, in the present study it was found that only attending religious meetings was associated with smoking behavior, in which it increased the probability of both spouses being non‐smokers.^29^

We did not find any association between the participants’ socioeconomic circumstances with spousal concordance on alcohol and tobacco consumption. One study that investigated socioeconomic factors and spousal concordance carried out in Brazil, found that non‐smoking concordance increased with higher levels of income and education, but their findings were related to adults aged 20 years and older with a mean age of 43.3 years, and not older adults only.^30^ The previously mentioned study carried out in Canada found that higher educational levels were associated with increased couple concordance in drinking habits.^22^ It seems that economic circumstances, including schooling, might influence couple concordance differently in relation to these health behaviors in different countries.

To the best of our knowledge, this is the first study to analyze health behavior concordance among older couples in low‐ and middle‐income countries. Even though we used the same protocol across the five country sites, few participants were found to be at‐risk drinkers or smokers, which limited our analysis. In addition, the cross‐sectional design prevented us inferring temporality regarding possible associations between health behavior concordance and their correlates, such as social network and drinking habits concordance. Another limitation was the secondary analysis nature of the present study, in which some information important to the present study was not collected (such as duration and quality of marriage, social roles, and the onset of some chronic conditions), limiting some of our analyses. Two recent studies using data from the Health and Retirement Study in the USA showed the importance drinking health behavior concordance has on the quality of marriage, an important factor affecting health in general, and also suggested that higher partner mastery belief (self‐efficacy) is associated with better self‐rated health and fewer functional limitations.^10^, ^12^

A common understanding between spouses regarding a need for change might be pivotal for the success of promoting healthy attitudes and behaviors. Future research using a longitudinal design aiming to deepen knowledge about the effect of each spouse's behavior on the other is required, especially in low‐ and middle‐income countries. The present findings show that group level interventions aimed at health behavior change might be more effective, and interventions targeting the family should be tested. Research in this field should also follow new family trends, as family patterns are changing, with higher divorce rates, changes in gender roles and married couples of the same gender.

## Disclosure statement

The authors declare no conflict of interest.
